# Developing and validating a risk assessment method for noise-induced hearing loss in workers

**DOI:** 10.1016/j.heliyon.2024.e40475

**Published:** 2024-11-17

**Authors:** Milad Abbasi, Saeid Yazdanirad, Ali Ahmadi

**Affiliations:** a Social Determinants of Health Research Center, Saveh University of Medical Sciences, Saveh, Iran; b Modeling in Health Research Center, Shahrekord University of Medical Sciences, Shahrekord, Iran; c Department of Occupational Health, School of Health, Shahrekord University of Medical Sciences, Shahrekord, Iran; d Modeling in Health Research Center and School of Public Health, Department of Epidemiology and Biostatistics, Shahrekord University of Medical Sciences, Shahrekord, Iran

**Keywords:** Risk assessment, Noise, Hearing loss, Structural equational modeling

## Abstract

Various risk factors can affect noise-induced hearing loss (NIHL) among employees. This research sought to establish and validate a risk assessment method for NIHL using these risk factors. This cross-sectional research was carried out with 220 workers from a steel manufacturing facility. At first, their demographic characteristics and information related to the studies’ items were gathered by the researcher-made questionnaire. In the next step, the noise exposure values of the participants were measured by the sound pressure level meter based on the ISO 9612 standard. Moreover, a pure tone audiometric test of bone and air conduction was used to measure hearing loss in people. Ultimately, a new index for risk assessment was created. Indirect effect coefficients of individual factors such as work experience, age, smoking, and effective diseases were 0.266, 0.227, 0.056, and 0.064, respectively. The coefficients of noise exposure items including occupational noise and leisure noise were obtained as 0.687 and 0.660. The personal protective equipment (PPE) items including use of PPE, noise reduction rating of PPE, and awareness of PPE were 0.194, 0.147, and 0.127, respectively. These coefficients were utilized to create a new index. The overall score of the index was divided into four categories using optimal cut-off values of 4.85, 6.84, and 7.59. According to the findings, the OHLRA methods were able to account for 74 percent of the hearing loss values. The results showed that the novel index for risk assessment had proper validity in the prediction of NIHL.

## Highlights

• Personal, noise exposure, and PPE factors had significant effect on the hearing loss.
• Highest effect coefficients were related to noise exposure factors.
• The validity of the novel index for risk assessment of hearing loss was acceptable.

## 1 Introduction

There are various adverse factors in the workplace that threaten human health [1,2]. Noise-induced hearing loss (NIHL) is caused by exposure to high-intensity sounds for prolonged periods a type of sensorineural hearing impairment [3]. Industrial NIHL represents a major issue in occupational health that affects workers in various industries [4]. According to the World Health Organization (WHO), approximately 1.1 billion adolescents and young adults around the globe are vulnerable to developing NIHL as a consequence of exposure to elevated noise levels exceeding 85 dB (dB) over prolonged periods [5]. In recent years, there have been several studies that have investigated the factors that contribute to NIHL. Exposure to excessive noise levels over time can lead to permanent harm to the auditory system, resulting in hearing impairment and other associated health issues [[6], [7], [8]]. Various aspects of noise exposure, such as the nature of the noise, the intensity and duration of exposure, and personal characteristics can all contribute to the onset of hearing impairment [9]. A study by Chen et al. examined the connection between hearing loss in workers and exposure to the occupational noise [10]. The study found that workers exposed to high levels of noise had a greater risk of developing hearing loss. Additionally, workers with longer exposure to noise were more likely to develop hearing loss compared to those with shorter exposure [10]. A study by Kirchner et al. discovered that workers exposed to noise levels exceeding 85 dB for more than 15 years exhibited a markedly higher probability of experiencing hearing loss [11]. The likelihood of NIHL rises with higher noise levels and longer exposure times. According to a recent study by Clifford et al., the type of exposure to industrial noise can affect the development of NIHL differently [12]. The study found that the likelihood of developing NIHL was higher among individuals exposed to impulse noise compared to continuous noise [13]. Personal factors, including genetic predisposition and age, have also been found to contribute to NIHL [14,15]. Age is a known factor that contributes to noise-induced hearing loss. A study by Abraham et al. investigated the incidence of NIHL among employees subjected to occupational noise. The study found that older workers exhibited a greater prevalence of NIHL in comparison to younger workers with similar levels of noise exposure [16]. Research carried out by AlQahtani et al. indicated that individuals with a familial background of hearing impairment exhibited a heightened vulnerability to NIHL [17]. Additional factors that may elevate the risk of NIHL include elevated blood pressure, smoking, and exposure to ototoxic chemicals [18]. A study by Li et al. found that smoking can have a significant impact on NIHL among industrial workers [19]. In this research, A positive relationship has been found between smoking and NIHL, indicating that the risk of developing NIHL increases with the amount of smoking [19]. In addition, workers with hypertension and/or diabetes who were subjected to elevated noise levels had a higher risk of developing NIHL than workers without these health conditions [20,21]. A study by Pelegrinet al. found that workers who used hearing protection devices had significantly lower rates of NIHL in contrast to individuals who did not utilize any protective measures [22]. Another research by Hong et al. examined the effectiveness of personal hearing protection in preventing NIHL in employees subjected to excessive levels of occupational noise [23]. The study found that both these devices were effective in reducing noise exposure and preventing NIHL [23]. Given the aforementioned risk factors and the significance of NIHL, it is essential to develop a risk assessment model that can determine the contribution of each factor towards the development of NIHL. Using structural equation modeling (SEM) it is possible to test complex relationships between variables and develop models that can predict the likelihood of an outcome. In the context of developing a risk assessment model for NIHL, SEM can be a powerful tool for identifying the key risk factors and their interrelationships. Consequently, the objective of the research was to establish and validate a risk assessment method for NIHL among workers, employing structural equation modeling.

## 2 Materials and methods

**Fig. 1:**
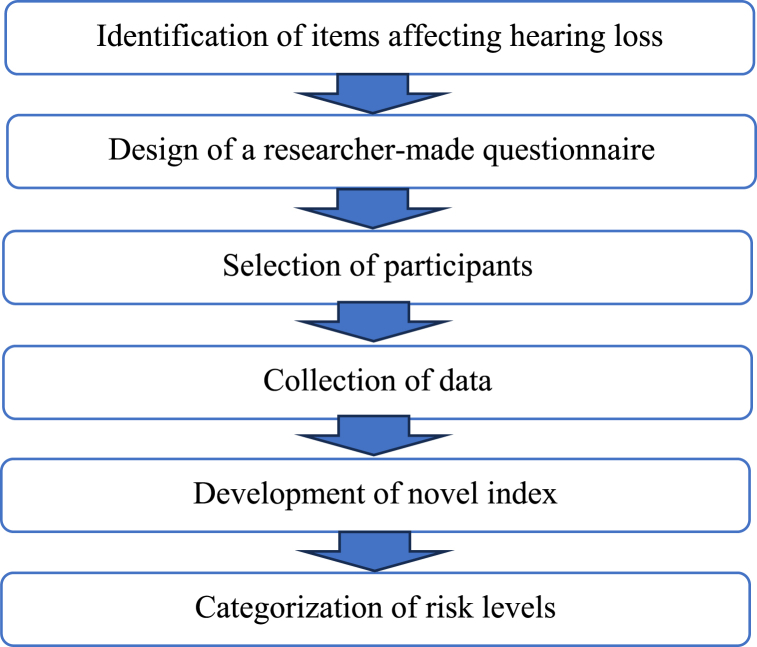
Flow chart of study steps.

**Fig. 2:**
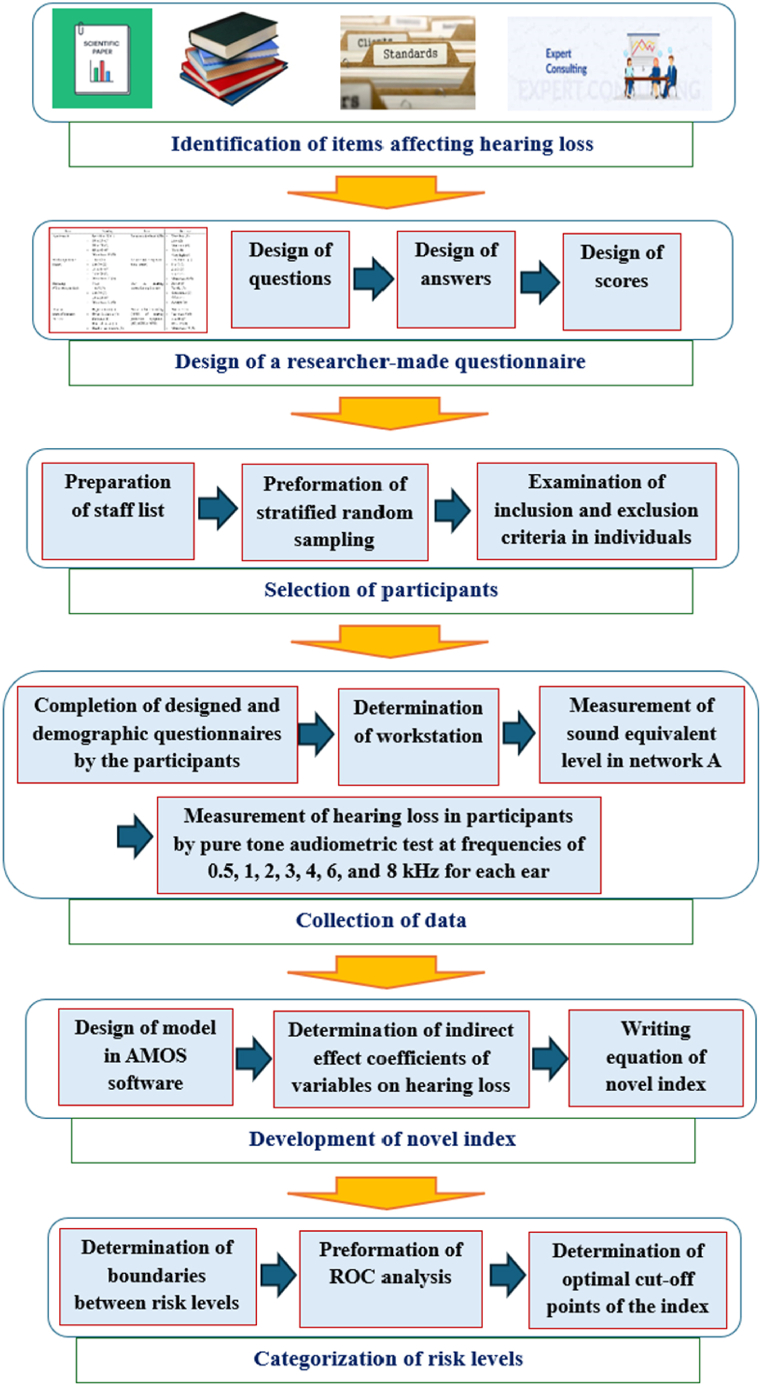
The detailed steps of the study.

The procedures of the study are illustrated in Fig. 1. Fig. 2 also depicts the detailed steps of this study.

### 2.1 Identification of items affecting hearing loss

At this stage, important items affecting hearing loss, such as age, work experience, smoking, use of hearing protection equipment, noise reduction rating of hearing protection equipment, and the level of noise exposure, were identified through a search of scientific articles, books, and standards, as well as consultations with experts in the field of noise. These items were then grouped based on similar characteristics.

### 2.2 Design of a researcher-made questionnaire

In this step, a researcher-made questionnaire was designed, including items related to age, work experience, smoking, diseases, occupational noise level, occupational noise exposure time, leisure noise level, leisure noise time, utilization of hearing protection devices, noise reduction rating of hearing protection equipment, and awareness of the use of hearing protection equipment. To assess the score associated with each personal item, a five-point Likert scale ranging from 1 to 5 was employed. Table 1 provides the scoring guidelines for the items under investigation.

**Table 1:** Instructions for categorizing personal items.

Item	Scoring	Item	Scoring
Age (years)	- Less than 20 (1) − 20 to 29 (2) − 30 to 39 (3) − 40 to 49 (4) - More than 49 (5)	Leisure noise level (dB)	- Very low (1) - Low (2) - Moderate (3) - High (4) - Very high (5)
Work experience (years)	− 1 to 5 (1) − 6 to 10 (2) − 11 to 15 (3) − 16 to 20 (4) - More than 20 (5)	Leisure noise exposure time (hours)	- Less than 1 (1) − 1 to 2 (2) − 2 to 3 (3) − 3 to 4 (4) - More than 4 (5)
Smoking (Cigarette per day)	− 0 (1) − 1 to 5 (2) − 6 to 10 (3) − 10 to 20 (4) - More than 20 (5)	Use of hearing protective equipment	- Never (1) - Rarely (2) - Sometimes (3) - Often (4) - Always (5)
Diseases (sum of diseases ‘scores)	- Hypertension (1) - Hyperlipidemia (1) - Diabetes (1) - Heart diseases (1) - Head or ear trauma (1)	Noise reduction rating (NRR) of hearing protective equipment (dB) (OSHA NRR)	- Non-use (5) - Less than 5 (4) − 5 to 10 (3) − 10 to 15 (4) - More than 15 (5)
Occupational noise level (dB)	- Less than 75 (1) − 75 to 80 (2) − 81 to 85 (3) − 86 to 90 (4) - More than 90 (5)	Awareness on use of hearing protective equipment	- Very low (5) - Low (4) - Moderate (3) - High (2) - Very high (1)
Occupational noise exposure time (hours)	- Less than 2 (1) − 2 to 4 (2) − 4 to 6 (3) − 6 to 8 (4) - More than 8 (5)		

### 2.3 Selection of participants

This cross-sectional study was conducted on 220 employees from various departments of a steel factory. Approval for the study was granted by the ethics committee with code number IR.SKUMS.REC.1401.037. Inclusion criteria were that employees had a regular presence at the factory, had no congenital hearing disorders or hearing disorders caused by accidents, had no second job, and had records of pre-employment and periodic examinations. Exclusion criteria included unwillingness to participate or cooperate during the study. Medical records and sound pressure level measurements from previous years were reviewed to select eligible workstations and individuals who met the inclusion criteria.

### 2.4 Collection of data

In this phase, individuals were initially invited to voluntarily participate in the study. Then, their demographic characteristics and information associated with to the study's items were gathered by the researcher-made questionnaire. In the next step, the exposure values of the participants were determined with a sound pressure level meter. To determine the noise exposure level of working people, the guidelines in the ISO 9612 standard were used [24]. For this purpose, the workstations of the participants were first determined. Then, at each position, the equivalent noise level (L_eq_) was determined as the amount of exposure in that position for 15 min by a sound level meter in network A [24]. Following this, the L_eq_ in Network A was determined grounded in the length of time spent at each station and the corresponding sound level measurements taken at those locations. Moreover, a pure tone audiometric test of bone and air conduction was used to measure hearing loss in participants. To perform the audiometric test, participant's hearing was evaluated using a clinical audiometer in a soundproof chamber in accordance with the standards set forth by the American Speech-Language-Hearing Association [25]. The pure tone hearing threshold of participants for each ear was measured by a skilled audiologist at frequencies of 500, 1000, 2000, 3000, 4000, 6000, and 8000 kHz. Before starting the study, the audiometer and all equipment were calibrated. Hearing loss was calculated by the mean value of threshold pure tone at frequencies of 500, 1000, 2000, and 3000 kHz in each ear.

### 2.5 Development of a novel index

Subsequent to constructing the model using AMOS software for risk assessment of hearing loss based on measured and determined variables, the values representing the indirect effects of the items on the alteration of hearing loss were utilized as variable coefficients (C1 to C9) in the novel index named occupational hearing loss risk assessment (OHLRA). The values of the variables also were the scores obtained from Table 2. This pattern has been used in previous studies for developing the equation of novel index [[26], [27], [28]]. The variables included age [16], work experience [29], smoking [30], diseases [[31], [32], [33], [34], [35]], occupational noise level [36], occupational noise exposure time [18], leisure noise level [37], leisure noise time [37], utilization of hearing protection devices [38], noise reduction rating of hearing protection devices [38], and awareness of the utilization of hearing protection devices [39]. Eq. (1) shows the equation used to compute the OHLRA.

Eq. (1) $$OHLRA=\left[\left(C_{1}\times A\right)+\left(C_{2}\times WE\right)+\left(C_{3}\times S\right)+\left(C_{4}\times D\right)+\left(C_{5}\times ON\right)+\left(C_{6}\times \mathrm{LN}\right)+\left(C_{7}\times UPPE\right)+\left(C_{8}\times NRR\right)+\left(C_{9}\times APPE\right)\right]$$

Where A is represents the score of age, WE is represents the score of work experience, S is represents the score of smoking, D is represents the score of diseases, ON is represents the score of occupational noise exposure, LN is represents the score of leisure noise exposure, UPPE is represents the score of utilization of PPE, NRR is represents the score of noise reduction rating of PPE, and APPE is represents the score of Awareness on PPE.

**Table 2:** Distribution statistics of the understidy variables.

Variable		Range	Mean	Standard deviation
Demographic parameters	Age (years)	20–58	35.31	7.92
	Work experience (year)	1–29	11.92	6.27
	Weight (kilogram)	29.00–146.00	81.48	13.85
	Height (meter)	1.54–1.93	1.75	0.07
	Body mass index (kg/m^2^)	16.98–34.72	25.27	3.36
	Smoking (number per day)	0–20	1.24	2.30
Personal items	Age	1–5	3.10	0.82
	Work experience	1–5	2.80	1.22
	Smoking	1–5	1.68	0.74
	Diseases	0–3	0.71	0.72
Noise exposure items	Occupational noise exposure	0.6–5	2.28	0.95
	Leisure noise exposure	0.2–5	1.80	1.33
Personal protective equipment items	Use	1–5	2.91	1.36
	Noise reduction rating	1–5	2.91	1.19
	Awareness	1–3	2.18	0.81
Hearing loss		10–67.26	31.46	17.95

### 2.6 Categorization of risk levels

In this stage, the overall score of the Occupational Hearing Loss Risk Assessment (OHLRA) was classified into four distinct risk categories, ranging from low to very high, through the analysis of Receiver Operating Characteristic (ROC) curves. In this ROC analysis, hearing loss thresholds of 25, 40, and 60 dB were identified as the demarcations between the risk categories [40]. To determine the optimal cut-off points for the index, the point closest to the ideal condition on each ROC curve was utilized.

### 2.7 Statistical analyses

Finally, the data were input into the Statistical Package for the Social Sciences (SPSS) version 26, where descriptive analyses were conducted. Following this, the normality of the variables was assessed through skewness and kurtosis analyses in AMOS. The findings indicated that all variables exhibited normal distributions. Consequently, correlations were computed using the Pearson correlation coefficient. In accordance with the findings, Drawing on the interconnections between the variables, an empirical model for evaluating the risk of occupational hearing loss was developed using AMOS software. The model's fit was assessed thourght three various type of fit indices: comparative, absolute, and normed [41]. Within the model, the factor loading values for the variables related to hearing loss (the gold standard variable) were determined. Subsequently, a new OHLRA index was created, and its scores were classified. To validate this index, a linear regression analysis was conducted to examine the relationship between the OHLRA scores and hearing loss values.

## 3 Results

Table 2 illustrates the statistical frequency and distribution of the demographic variables under investigation. The results revealed that individuals with various noise exposure participated in the study. The hearing loss value also exhibited a broad range, spanning from 10 to 67.26 dB.

**Fig. 3:**
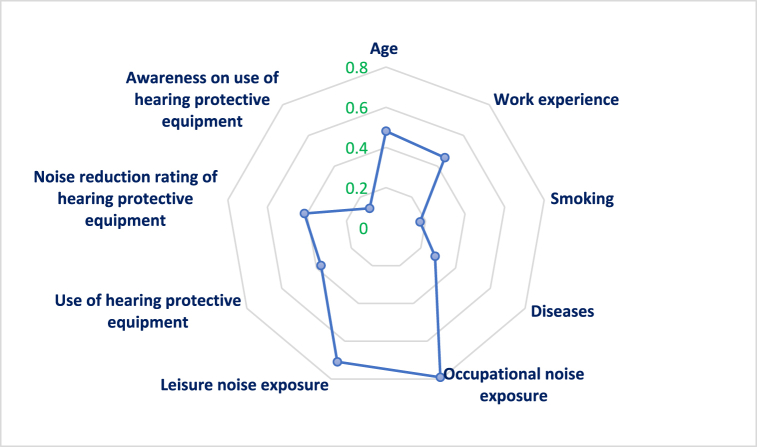
The correlation coefficients of the variables in contributing to hearing loss.

Table 3 and Fig. 3 present the reletionship between the items and the hearing loss values. The findings indicate significant correlations between all items and the occupational hearing loss score (P < 0.01). Among individual variables, age (0.481) exhibited the strongest correlation with the hearing loss value. Among the noise exposure factors, the most substantial correlations were associated with the variable of occupational noise exposure (0.761). Among hearing protective equipment, the noise reduction rating (NRR) of hearing protective equipment had the highest correlation with the hearing loss value (0.413).

**Table 3:** The correlation coefficient between the items and hearing loss value.

Parameter		1	2	3	4	5	6	7	8	9	10
1	Age	–									
2	Work experience	0.778∗∗	–								
3	Smoking	0.286∗∗	0.230∗∗	–							
4	Diseases	0.208∗∗	0.169∗	0.365∗∗	–						
5	Occupational noise	0.454∗∗	0.438∗∗	0.203∗∗	0.238∗∗	–					
6	Leisure noise	0.330∗∗	0.337∗∗	0.107	0.147∗	0.740∗∗	–				
7	PPE Use	0.129	0.085	0.179∗∗	0.094	0.201∗∗	0.260∗∗	–			
8	NRR	0.202∗∗	0.176∗	0.039	0.220∗∗	0.336∗∗	0.378∗∗	0.196∗∗	–		
9	Awareness	0.186∗∗	0.158∗	0.169∗	0.196∗∗	0.280∗∗	0.265∗∗	0.350∗∗	0.258∗∗	–	
10	Hearing loss	0.481∗∗	0.456∗∗	0.172∗	0.282∗∗	0.791∗∗	0.709∗∗	0.374∗∗	0.413∗∗	0.327∗∗	–

∗P < 0.05, ∗∗P < 0.001.

**Fig. 4:**
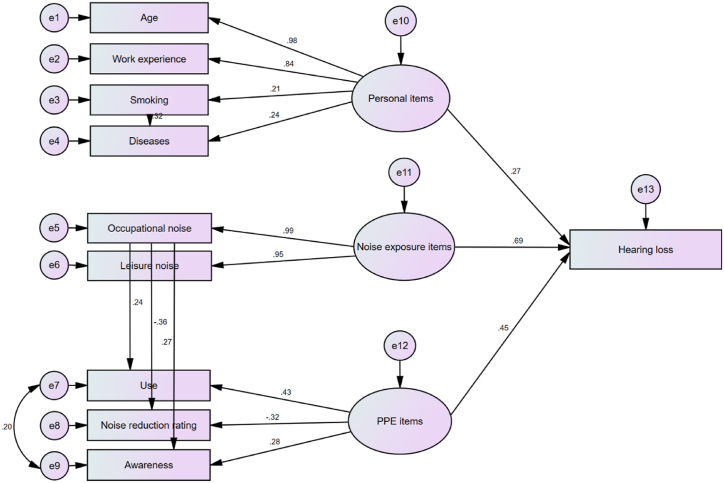
The theoretical model.

Fig. 4 illustrates the proposed conceptual framework. The findings indicated that personal items yielded a total coefficient of 0.271, while items associated with noise exposure produced a total coefficient of 0.691. Additionally, hearing protection equipment items was linked to a total coefficient of 0.452, all of which demonstrated a significant influence on the degree of hearing loss.

**Fig. 5:**
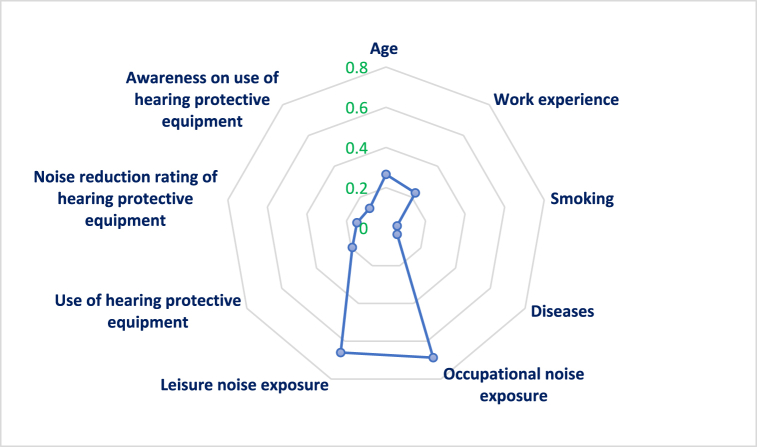
Coefficients representing the indirect effects of various variables associated with the development of NIHL.

Table 4 and Fig. 5 illustrate the impact coefficients of various factors on the incidence of hearing impairment. Among the personal attributes, age (0.266) and work experience (0.227) exhibited the most significant indirect effect coefficients on hearing loss, respectively. Among the factors related to noise exposure, the variable with the most substantial indirect effects was occupational noise exposure (0.687). Among the items related to hearing protective equipment, the utilization of such equipment demonstrated the strongest correlation with the incidence of hearing loss (0.194).

**Table 4:** Coefficients of the variables influencing hearing loss.

Variable		Effects		P-value
		Direct	Indirect	
Personal items	Age	0.980	0.266	<0.001
	Work experience	0.837	0.227	<0.001
	Smoking	0.207	0.056	0.014
	Diseases	0.236	0.064	0.009
	Total	0.271	–	0.002
Noise exposure items	Occupational noise exposure	0.994	0.687	0.001
	Leisure noise exposure	0.955	0.660	0.001
	Total	0.691	–	0.001
Personal protective equipment items	Use	0.429	0.194	0.008
	Noise reduction rating	0.325	0.147	0.004
	Awareness	0.280	0.127	0.005
	Total	0.452	–	0.009

Table 5 presents the goodness-of-fit indices for the analyzed model, confirming its adequacy. The indirect effect coefficients of the variables were employed to formulate the OHLRA equation as follows:

Eq. (2) OHLRA=[(0.266×A)+(0.227×WE)+(0.056×S)+(0.064×D)+(0.687×ON)+(0.660×LN)+(0.194×UPPE)+(0.147×NRR)+(0.127×APPE)]

**Table 5:** Fit indices for the evaluated model.

index	Name	Fitness Benchmark	Achieved Metric
Absolute	RMSEA a	Less than 0.1	0.087
	X^2^/df b	1–3	2.597
Comparative	GFI c	More than 0.9	0.942
	AGFI d	More than 0.9	0.933
Normed	NFI e	More than 0.9	0.936
	CFI f	More than 0.9	0.959
	IFI g	0 to 1	0.960

a Root mean squared error of approximation.

b .Normed Chi-square.

c .Goodness-of-fit index.

d .Adjusted goodness-of-fit index.

e .Normed fit index.

f .Comparative fit index.

g .Incremental fit index.

The scores of occupational noise and leisure noise variables are computed as follows:

Eq. (3) $$ON=\frac{\left(t_{ON}\times L_{ON}\right)}{5}$$

Where $t_{ON}$ is the score of duration of exposure to noise occupational, and $L_{ON}$ is the score of level of occupational noise exposure.

Eq. (4) $$\mathrm{LN}=\frac{\left(t_{\mathrm{LN}}\times L_{\mathrm{LN}}\right)}{5}$$

Where $t_{\mathrm{LN}}$ is the score of duration of leisure noise exposure, and $L_{\mathrm{LN}}$ is the score of level of leisure noise exposure.

**Fig. 6:**
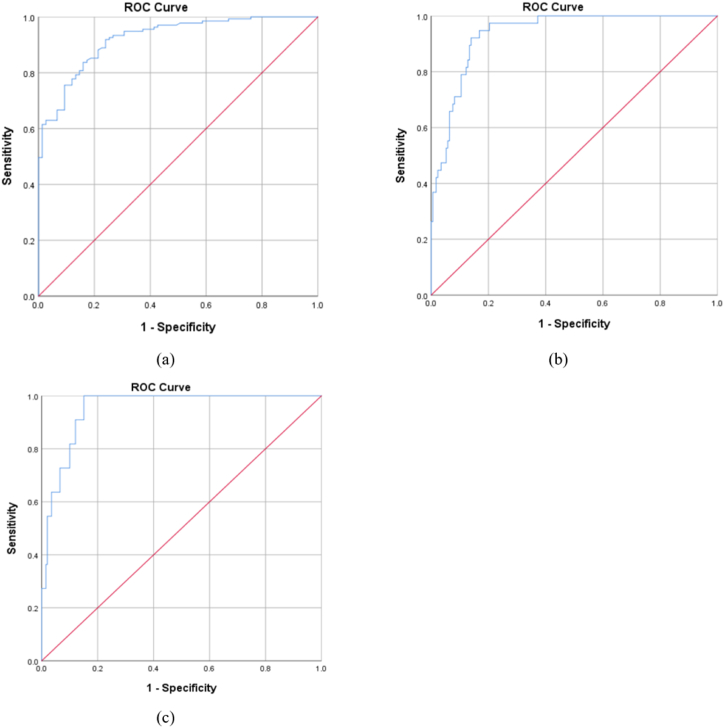
ROC curves for risk areas: (a) low versus moderate risk, (b) moderate versus high risk, and (c) high versus very high risk.

**Fig. 7:**
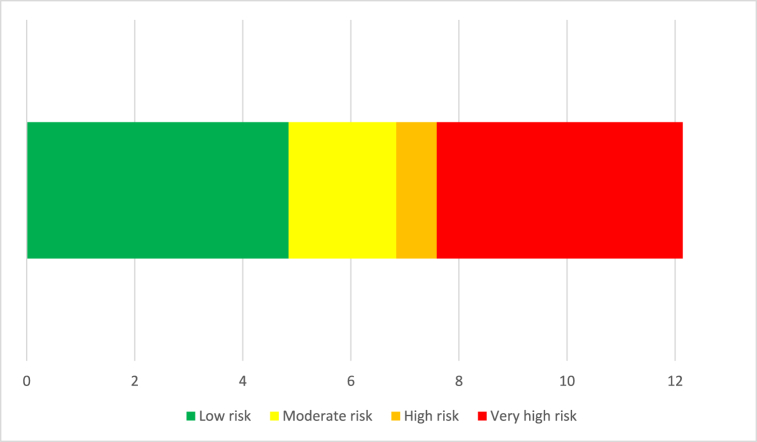
The risk levels of the novel index.

The receiver operating characteristic (ROC) curves is presented in Fig. 6(a–c). The ideal cut-off thresholds distinguishing low from moderate risk, moderate from high risk, and high from very high risk were determined to be 4.85 (specificity = 0.840, sensitivity = 0.837), 6.84 (specificity = 0.860, sensitivity = 0.921), and 7.59 (specificity = 0.879, sensitivity = 0.909), respectively. The area under the curves (AUC) was calculated to be 0.922 (95 % CI: 0.887, 0.957) (p < 0.001), 0.937 (95 % CI: 0.904, 0.970) (p < 0.001), and 0.952 (95 % CI: 0.916, 0.988) (p < 0.001), respectively. The various risk levels along with their corresponding OHLRA scores are detailed in Table 6 and illustrated in Fig. 7. Based on the results, the OHLRA method could account for 74 percent of the hearing loss values.

**Table 6:** Risk categories corresponding to OHLRA evaluation scores.

Risk Categories	corresponding score for OHLRA
Very high	≤7.59
High	6.84 to 7.59
Moderate	4.85 to 6.83
Low	≥4.85

## 4 Discussion

The objective of this investigation was to establish and validate a method for assessing the risk level of NIHL in workers using SEM. The researchers applied a correlation analysis to specify the relationship between the different items and the NIHL value. The findings demonstrated a strong correlation between all items and the NIHL score. Specifically, age, occupational noise exposure, and noise reduction rating (NRR) were found to have the greatest relationship with the NIHL value among the personal characteristic, noise exposure items, and hearing protective equipment items, respectively.

Based on the available literature, age has been found to have a significant impact on the occurrence of NIHL, particularly in high frequencies [12]. Additionally, studies have highlighted the importance of individual susceptibility along with noise exposure [42]. Various agent, including smoking, changes in lipid metabolism, and salicylate intake have been recognized as having a collaborative influence on the occurrence of NIHL [[42], [43], [44]]. Based on the results presented in Tables 3 and it is evident that age, experience, smoking, and disease are significantly related to hearing loss. Additionally, Table 4 highlights that age has a greater impact on hearing loss, as experience, smoking, and disease contribute to their effects through age. As individuals age, diseases like diabetes or cardiovascular disorders tend to increase [45,46] and can potentially contribute to hearing loss either synergistically or additively [33,47,48]. However, at times the severity of these diseases may be low and their effects may not be detectable, thus being attributed to the natural aging process. The findings display that work experience has a significant direct and indirect influence on hearing loss. It is logical to explain that as work experience increases, an individual's cumulative exposure to excessive noise levels also increases, which can bring about hearing loss. In line with the current investigation, Karimi et al. conducted a study that informed a substantial relationship among hearing loss and working experience, specifically in the frequencies of 500, 1000, 2000, and 4000 Hz [49]. Exposure to excessive noise levels in certain occupations can bring about an increase in occupational hearing loss as work experience accumulates over time. In addition to age and work experience, health conditions can have a substantial influence on the occurrence of hearing loss. The results indicated that having a disease can contribute to an increase in the hearing threshold level. In other words, many diseases and disorders have a detrimental effect on hearing. Previous studies have shown that diseases such as Alzheimer's disease, diabetes mellitus, cardiovascular disease, hypertension, Meniere's disease, and endocrine disorders can have a considerable effect on hearing loss [[50], [51], [52], [53], [54]]. According to the findings in Table 3, Table 4, age, work experience, disease, and smoking were found to be related to hearing loss. These individual factors accounted for 27.1 % of the changes in hearing loss, highlighting their significance in predicting hearing loss. This statement highlights the complex interplay between various factors that can contribute to hearing loss, as they can mutually influence and amplify each other's effects. This statement highlights the complex interplay between various factors that can contribute to hearing loss, as they can mutually influence and amplify each other's effects.

According to the results, noise exposure was shown to be the most substantial variable affecting hearing loss. This aligns with prior studies indicating that significant exposure to elevated levels of noise in occupational and recreational settings may result in auditory deficits [55,56]. Notably, in the present study, occupational noise exposure was found to have a greater impact than leisure noise. This may be ascribed to the circumstances that the understudy population were primarily workers who were more likely to be subjected to workplace noise rather than leisure noise. The results from the investigation carried out by Neitzel et al. suggest that the criteria used to assess occupational noise can also be applied to recreational noise [55]. This can be attributed to the absence of noteworthy distinctions detected between these two types of noise in terms of their impact on hearing. The indirect influence of noise exposure on the development of hearing impairment was determined to be markedly substantial. Referring to Fig. 1, noise exposure as an environmental stressor can prompt individuals to seek ways to reduce or avoid noise, increasing their awareness of the issue. Additionally, those who are exposed to noise may choose to use noise protection equipment, which can have a substantial impression on their hearing health. The results suggest that noise exposure accounts for approximately 69.1 % of hearing loss incidents, whether it is due to occupational exposure, leisure noise, or a combination of both.

Based on the findings, personal protective equipment items were shown to have a significant effect on preventing hearing loss. The results suggest that the utilization of noise protection equipment, the noise reduction rating of the devices, and awareness of NIHL, were associated with hearing loss. Together, these factors were able to predict 45.2 % of hearing loss cases. The results suggest that the use of noise protection devices was more important than either the noise reduction rating of the devices or awareness of noise-induced hearing loss in preventing hearing loss. A research by Alnuman et al. found that labors are rather likely to use hearing loss protection devices when they have knowledge about hearing loss [57]. Similarly, Dieck et al. reported high awareness and positive attitudes toward hearing loss [58]. Improving knowledge about hearing loss can encourage workers to utilize hearing protection devices and prevent hearing loss [57,59]. Even though having knowledge about hearing loss is vital, it may not always motivate people to use hearing protection devices due to their negative attitudes toward them. Some people may view these devices as unattractive or strange [57].

The findings of this study specify that noise exposure parameters exert a more significant influence on hearing loss compared to personal protective equipment and personal items [10,12,55]. This highlights the significant role of noise exposure in hearing loss occurrence, particularly in occupational settings. Additionally, exposure to loud noise can worsen the aging process, and the combination of aging and noise exposure can accelerate hearing loss [60]. Contrary to individual factors, personal protective equipment items have a more substantial impact on the occurrence of hearing loss. This result is expected because the general population is more likely to experience age-related hearing loss, whereas NIHL is less likely since they are not routinely subjected to industrial noise. As this study was conducted among industrial employees subjected to elevated noise levels, it is reasonable to expect that the utilization of hearing protection devices with a high noise reduction rate would have a greater effect than individual items such as age in preventing hearing loss. The results of this study can be considered highly valid as the area under the ROC curves was estimated to be high for all cut-off points. Additionally, the method used in this study was able to explain 74 % of the hearing loss values, further indicating its validity. The findings of this research demonstrate that individual characteristic, exposure to noise, and the utilization of personal protective devices significantly influence hearing loss. These findings hold substantial value for society, industry, and workers. By highlighting the critical role of exposure to the industrial noise, the research underscores the need for enhanced safety measures and regulations in workplaces. The insights gained can inform industry practices, leading to better training programs and the execution of efficacious auditory protection measures. Furthermore, raising awareness among workers about the dangers linked to exposure to noise and the importance of using PPE can empower them to take proactive steps in protecting their hearing. Ultimately, these results can guide future developments in occupational health policies and contribute to creating safer work environments, thereby improving overall worker well-being and productivity.

This study has some limitations that do not affect its validity but they may impact the generalizability of the results. Thus, researchers should take these restrictions into account in future studies to ensure the findings can be applied to a wider population.

There are several factors that are not considered in the current model for hearing loss. Firstly, heredity is a significant influencing factor for hearing loss, particularly age-related hearing loss, but it is not included in the model. Additionally, noise sensitivity, which is dependent on heredity, may also impact hearing loss but is not accounted for. Secondly, the frequency composition and temporal envelope of noise are main features that govern the severity and variety of NIHL, but they are not considered in the model. Finally, co-exposure to toxic materials such as metals and solvents, as well as the use of certain medications and vitamins/supplements like N-acetylcysteine, antibiotics, folic acid, and B12 which can have harmful or protective effects on the auditory system, are not considered in the model [61].

## 5 Conclusion

This study introduces a novel index for risk assessment that enhances our understanding of NIHL by integrating personal factors, noise exposure variables, and the utilization of hearing protection equipment. Unlike existing research, this model employs structural equation modeling to elucidate the complex relationships among these factors, offering a more comprehensive analysis of their impact on hearing loss. The findings indicate that noise exposure is the most significant cause of occurring hearing loss, emphasizing the urgent requirement for proper regulation of workplace noise levels. Individual factors, such as age, work experience smoking and disease, also play important roles, suggesting that personal characteristics should be considered in risk assessments. Furthermore, the study underscores the role of hearing protection equipment, emphasizing their potential in mitigating the risk of hearing loss. By providing a holistic view of the contributing variables, our model not only validates the significance of these factors but also serves as a valuable framework for future research and practical applications in occupational health. This comparative perspective situates our findings within the broader context of existing literature, reinforcing the unique contributions of our study to the field of risk assessment for NIHL.

## CRediT authorship contribution statement

**Milad Abbasi:** Writing – original draft, Visualization, Methodology, Investigation, Data curation, Conceptualization. **Saeid Yazdanirad:** Writing – review & editing, Supervision, Project administration, Methodology, Investigation, Data curation, Conceptualization. **Ali Ahmadi:** Writing – original draft, Visualization, Formal analysis.

## Ethics and consent

This study was approved as a research project by the ethics committee of Shahrekord University of Medical Sciences with code number IR.SKUMS.REC.1401.037. All methods were performed in accordance with relevant guidelines and regulations. Informed consent was acquired from the participants and that the participants consented to the publishing of all clinical data and other data included in the manuscript.

## Availability of data and materials

The datasets used and/or analyzed during the current study are available from the corresponding author on reasonable request.

## Funding

This study was supported by 10.13039/501100005756Shahrekord University of Medical Sciences.

## Declaration of competing interest

The authors declare that they have no known competing financial interests or personal relationships that could have appeared to influence the work reported in this paper.
